# Author Correction: A hollow-tube-like hydrospongel for multimodal therapy of advanced colorectal cancer

**DOI:** 10.1038/s41467-026-68519-9

**Published:** 2026-01-17

**Authors:** Tao Wu, Tenghui Li, Chengzhi Zhang, Yu Tian, Hao Li, Yuxin He, Xu Yan, Tianxing Gong, Junhua Zhao, Zhenning Wang

**Affiliations:** 1 Department of Biomedical Engineering, Shenyang University of Technology, Shenyang, China; 2 College of Medicine and Biological Information Engineering, Northeastern University, Shenyang, China; 3 Department of Surgical Oncology and General Surgery, The First Hospital of China Medical University, Shenyang, China; 4 Key Laboratory of Precision Diagnosis and Treatment of Gastrointestinal Tumors, Ministry of Education, China Medical University, Shenyang, China; 5 Department of Critical Care Medicine, The First Hospital of China Medical University, Shenyang, China; 6 The VIP Department, School and Hospital of Stomatology, China Medical University, Liaoning Provincial Key Laboratory of Oral Diseases, Shenyang, China

**Keywords:** Colorectal cancer, Biomedical materials, Chemotherapy

Correction to: *Nature Communications* 10.1038/s41467-025-62880-x, published online 12 August 2025

In the version of this article initially published, due to figure preparation mistakes, incorrect images were presented in Fig. 5g (HSP70/Group 6), Fig. 7g (Cleaved caspase 3/Group 6 and HSP70/Group 3), Supplementary Fig. 24 (Heart/Group 4) and in Supplementary Fig. 31 (Heart/Group 6). The errors do not affect the results of the study. The figures have been updated in the HTML and PDF versions of the article.

