# Supplementary material for: A hollow-tube-like hydrospongel for multimodal therapy of advanced colorectal cancer
Source: Nat Commun. 2025 Aug 12;16:7464. doi: 10.1038/s41467-025-62880-x (PMC12343928; doi:10.1038/s41467-025-62880-x)
Supplement: Supplementary file 1 — Supplementary Information [file 41467_2025_62880_MOESM1_ESM.pdf]

Supplementary Information

A Hollow-Tube-Like Hydrospongel for Multimodal Therapy of Advanced Colorectal Cancer

Tao Wu[1,2†], Tenghui Li[3,4†], Chengzhi Zhang[3,4], Yu Tian[1], Hao Li[1], Yuxin He[5], Xu Yan[6], Tianxing Gong[1*], Junhua Zhao[3,4*], Zhenning Wang[3,4*]

[1]Department of Biomedical Engineering, Shenyang University of Technology; Shenyang, China.

[2]College of Medicine and Biological Information Engineering, Northeastern University; Shenyang, China.

[3]Department of Surgical Oncology and General Surgery, The First Hospital of China Medical University; Shenyang, China.

[4]Key Laboratory of Precision Diagnosis and Treatment of Gastrointestinal Tumors, Ministry of Education, China Medical University; Shenyang, China.

[5]Department of Critical Care Medicine, The First Hospital of China Medical University; Shenyang, China.

[6]The VIP Department, School and Hospital of Stomatology, China Medical University, Liaoning Provincial Key Laboratory of Oral Diseases; Shenyang, China

[*]Corresponding author Email: znwang@cmu.edu.cn (Z.N.W.); tianx.gong@gmail.com (T.X.G.); jhzhao@cmu.edu.cn (J.H.Z.).

†These authors contributed equally to this work.

Table of Contents

Supplementary Figures 3

Supplementary Tables 16

Supplementary Figures

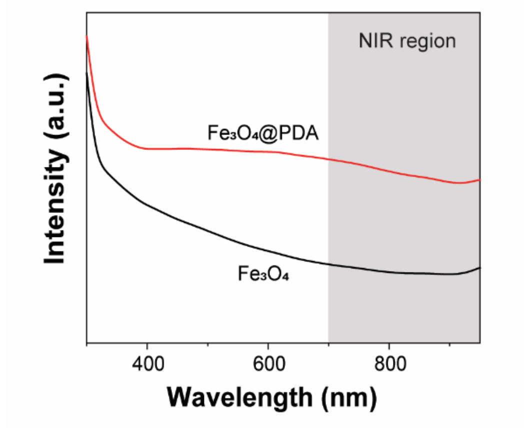

Supplementary Fig. 1 | Synthesis and characterization of Fe_3O_4 and Fe_3O_4@PDA NPs. The UV-Vis spectra of Fe_3O_4 and Fe_3O_4@PDA NPs in aqueous solution.

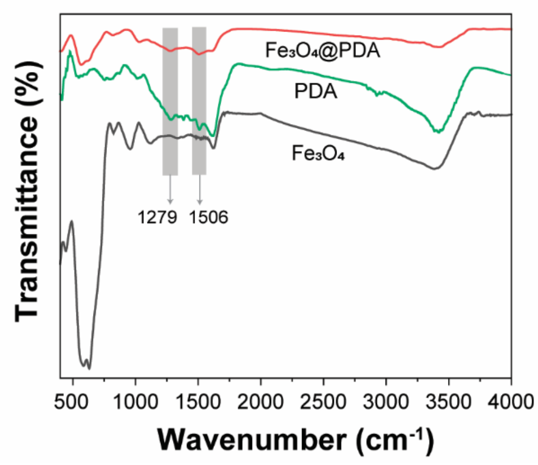

Supplementary Fig. 2 | Synthesis and characterization of Fe_3O_4 and Fe_3O_4@PDA NPs. The FTIR spectra of Fe_3O_4 and Fe_3O_4@PDA NPs.

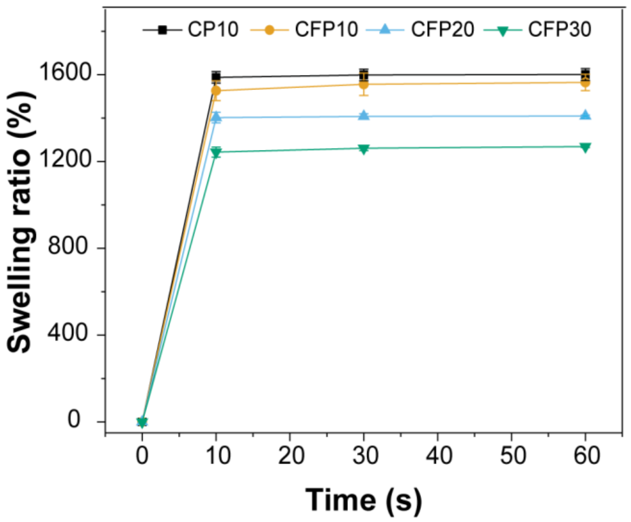

Supplementary Fig. 3 | Synthesis and characterization of HTHSG. Swelling behavior of hydrospongels in 60 s. The data were presented as the mean ± SD (n = 5 independent samples).

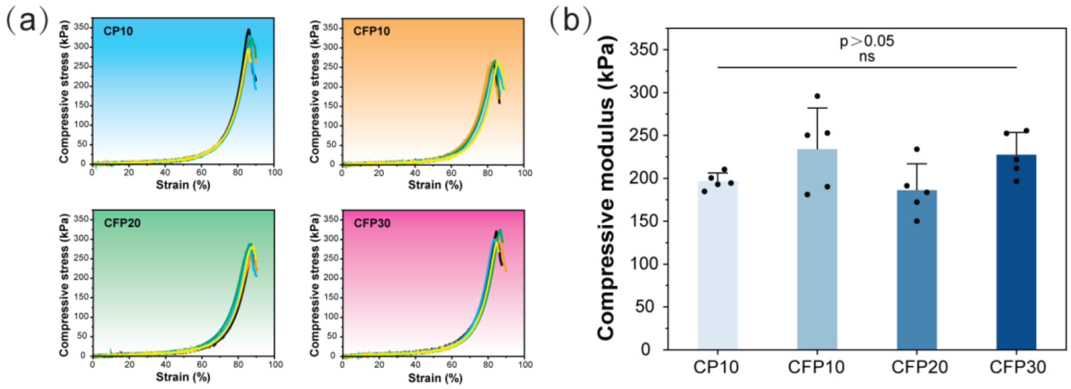

Supplementary Fig. 4 | Synthesis and characterization of HTHSG. **a,** The compressive stress-strain curves of the swollen hydrospongels (n = 5 independent samples). **b**, Compressive modulus of the swollen hydrospongels. The data were presented as the mean ± SD (n = 5 independent samples). Statistical differences were analyzed by one-way ANOVA first, and then by the Tukey's post hoc test. (ns, no significance).

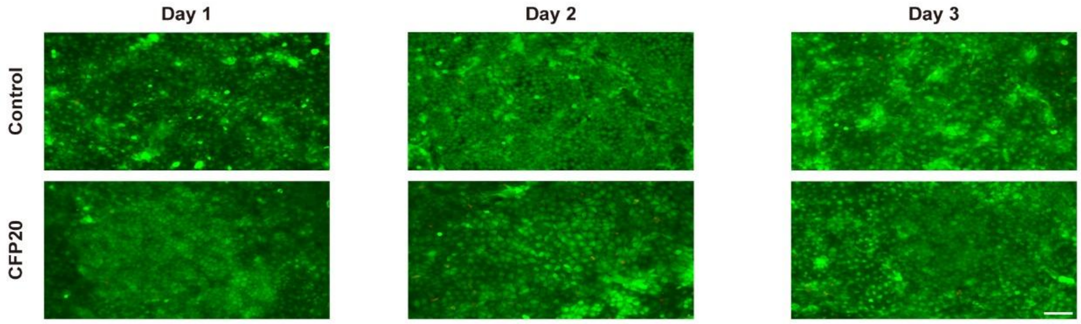

Supplementary Fig. 5 | Synthesis and characterization of HTHSG. Representative images of Calcein-AM (green) and propidium iodide (PI, red) stained NCM 460 cells to detect cellular viability across control and CFP20 groups. Scale bars: 100 μm. Each experiment was repeated five times independently with similar results.

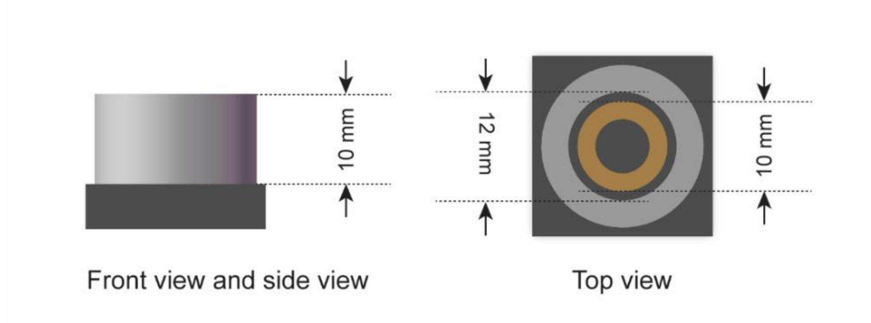

Supplementary Fig. 6 | Synthesis and characterization of HTHSG. Detailed dimensions of the 3D-printed mold.

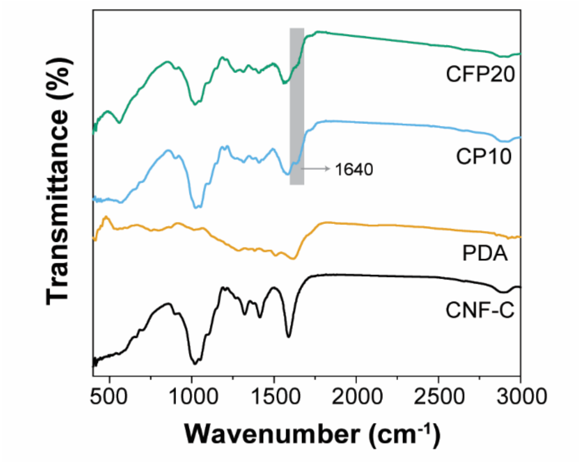

Supplementary Fig. 7 | Synthesis and characterization of HTHSG. FTIR spectra of CNF-C, PDA, CP10, and CFP20.

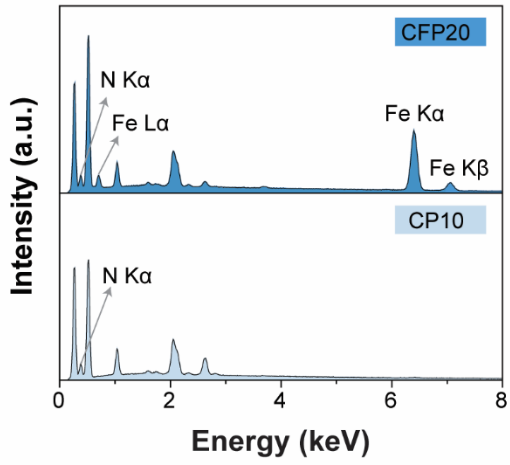

Supplementary Fig. 8 | Synthesis and characterization of HTHSG. The EDS spectra of CP10 and CFP20.

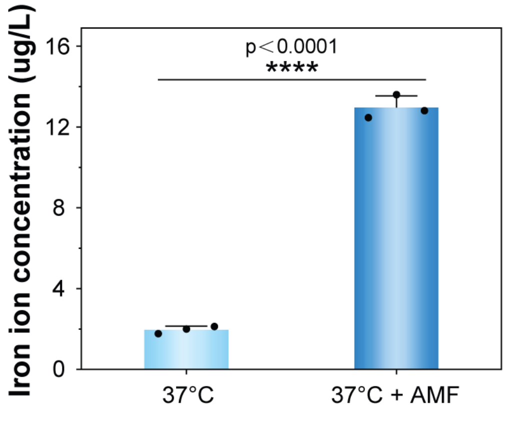

Supplementary Fig. 9 | Synthesis and characterization of HTHSG. The concentration of iron ions in different mediums after 7 days. The data were presented as the mean ± SD (n = 3 independent samples). Statistical differences were analyzed by two-tailed unpaired Student's t-test. (****$p < 0.0001$).

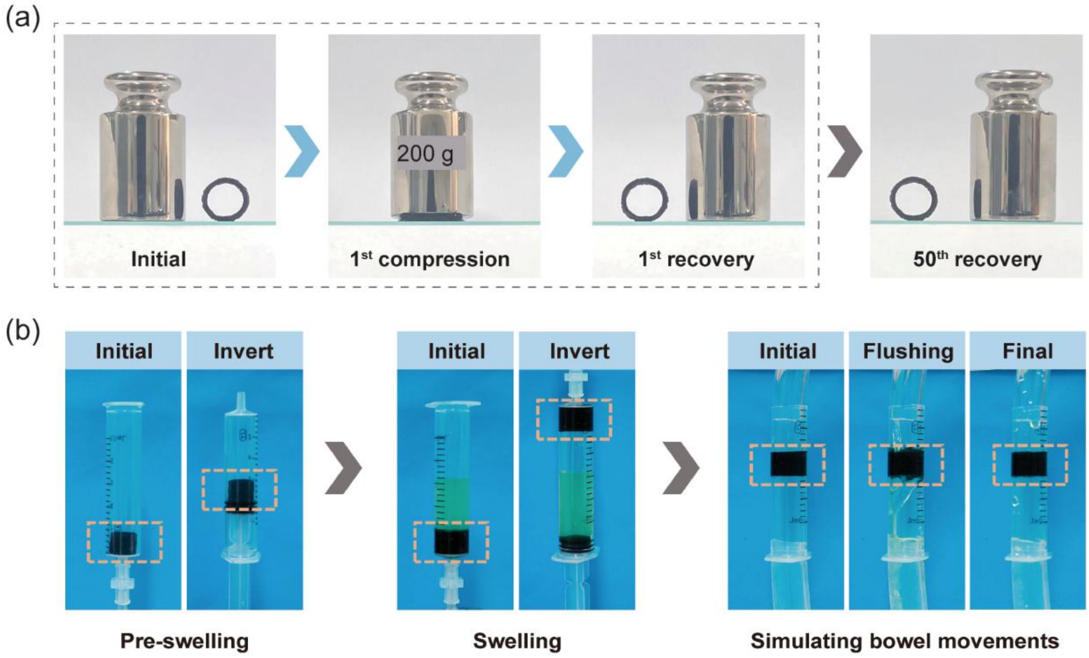

Supplementary Fig. 10 | Structural integrity and stability of HTHSG. **a,** Images of the swollen CFP20 hollow tube before and after compression by weights. **b,** Images of the CFP20 hollow tube in pre-swelling, swelling, and simulating bowel movements.

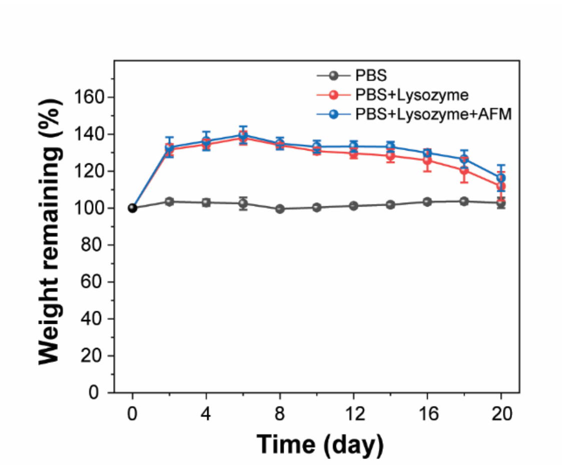

Supplementary Fig. 11 | Structural integrity and stability of HTHSG. Degradation behavior of CFP20 hollow tube under different conditions over 20 d. The data were presented as the mean ± SD (n = 5 independent samples).

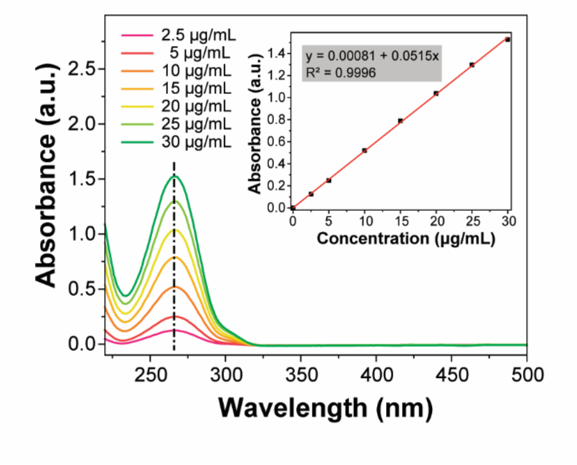

Supplementary Fig. 12 | Structural integrity and stability of HTHSG. UV-Vis spectra of different 5-FU solutions and the standard curve fitted according to the absorbance at 266 nm (insert).

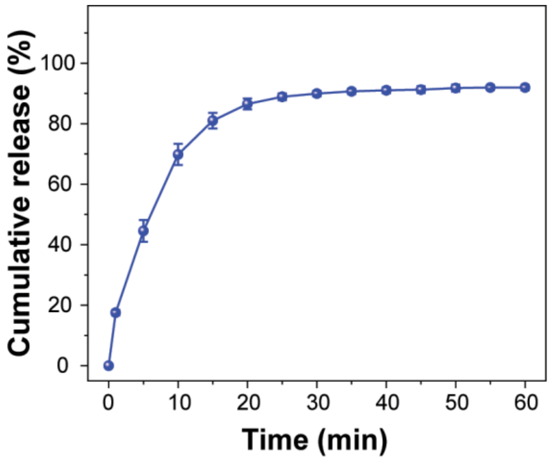

Supplementary Fig. 13 | Structural integrity and stability of HTHSG. The release profile of 5-FU from the CFP20 hollow tube within 60 min. The data were presented as the mean ± SD (n = 4 independent samples).

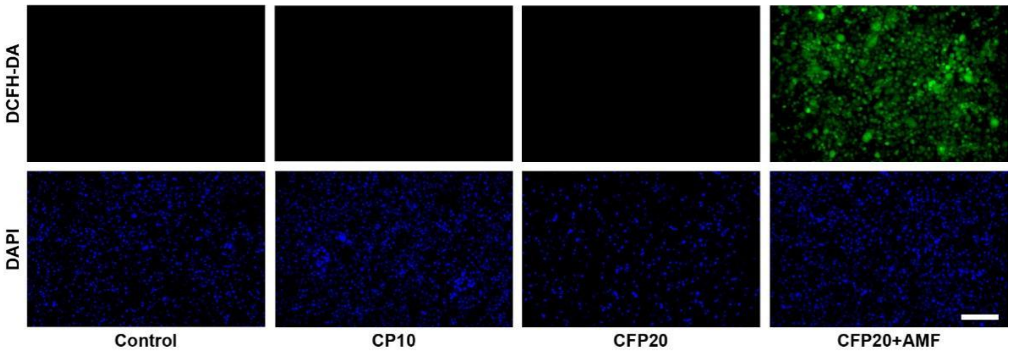

Supplementary Fig. 14 | In vitro anti-tumor effect of HTHSG. Representative single-color images of DAPI and DCFH-DA stained HCT-116 cells to detect the intracellular •OH across control, CP10, CFP20, and CFP20+AMF groups. Scale bars: 100 μm. Each experiment was repeated five times independently with similar results.

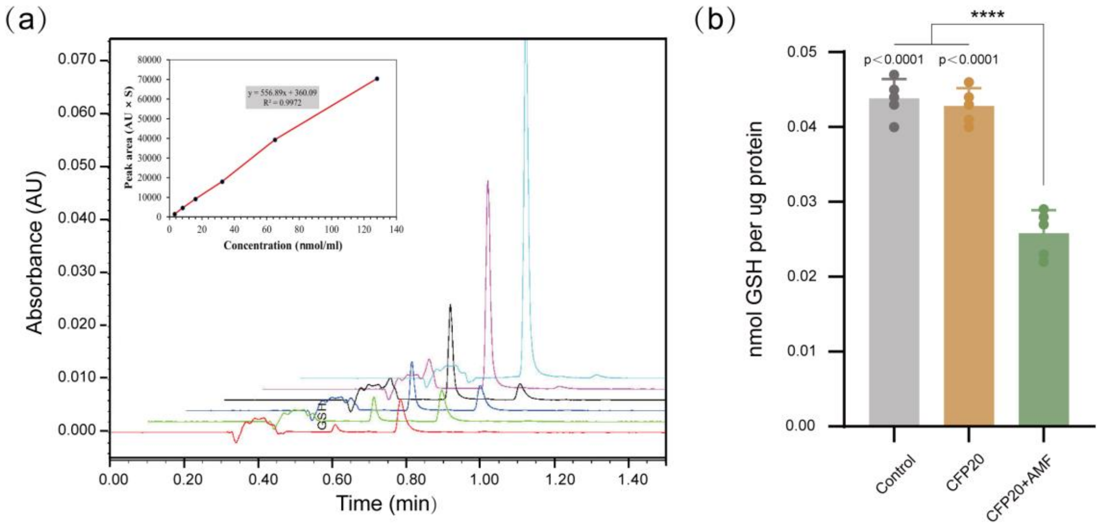

Supplementary Fig. 15 | In vitro anti-tumor effect of HTHSG. **a,** UPLC-PDA spectra of GSH solutions from 1μg/mL (3.2 nmol/mL) to 40 μg/mL (128 nmol/mL) and the standard curve fitted according to the absorbance at 412 nm (insert). **b,** GSH amount in HCT-116 cells after different treatments. The data were presented as the mean ± SD (n = 5 independent experiments). Statistical differences were analyzed by one-way ANOVA first, and then by the Tukey's post hoc test. (****$p < 0.0001$).

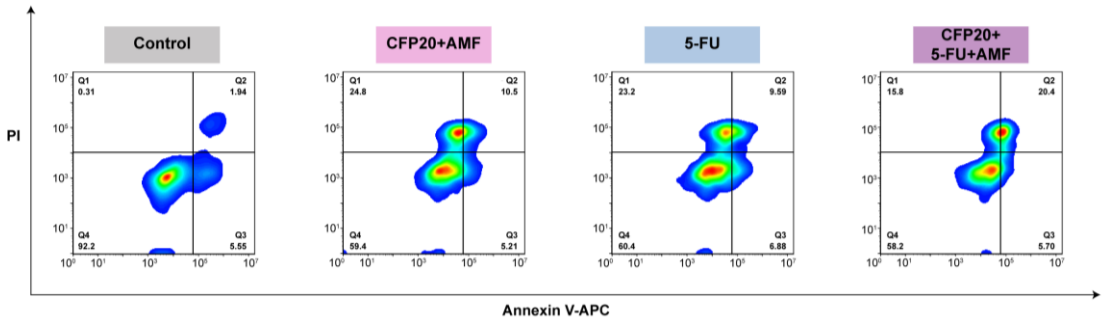

Supplementary Fig. 16 | In vitro anti-tumor effect of HTHSG. Apoptosis of HCT-116 cells in the control, CFP20+AMF, 5-FU, and CFP20+5-FU+AMF groups was analyzed by Annexin V-APC and PI staining.

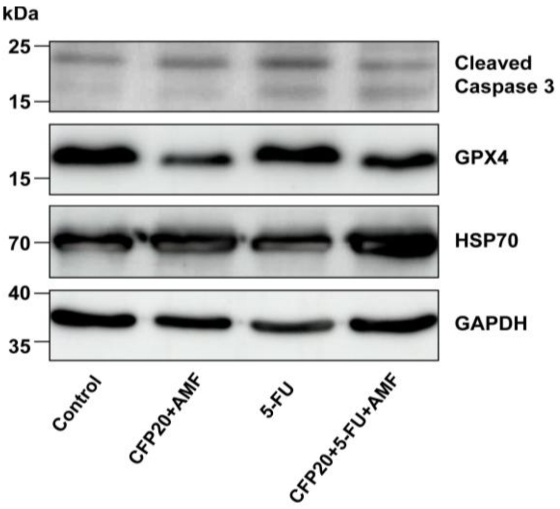

Supplementary Fig. 17 | In vitro anti-tumor effect of HTHSG. Western blotting assay of intracellular Cleaved Caspase 3, GPX4, and HSP70 expression in the control, CFP20+AMF, 5-FU, and CFP20+5-FU+AMF groups.

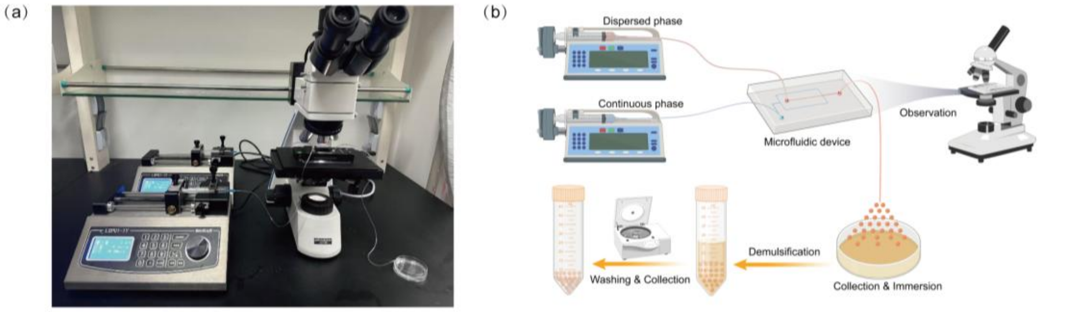

Supplementary Fig. 18 | In vitro anti-tumor effect of HTHSG. **a,** The digital photograph of the experimental setup for the preparation of drug-loaded hydrogel microspheres. **b,** The schematic diagram of the preparation process of drug-loaded hydrogel microspheres. This figure is created with MedPeer (medpeer.cn).

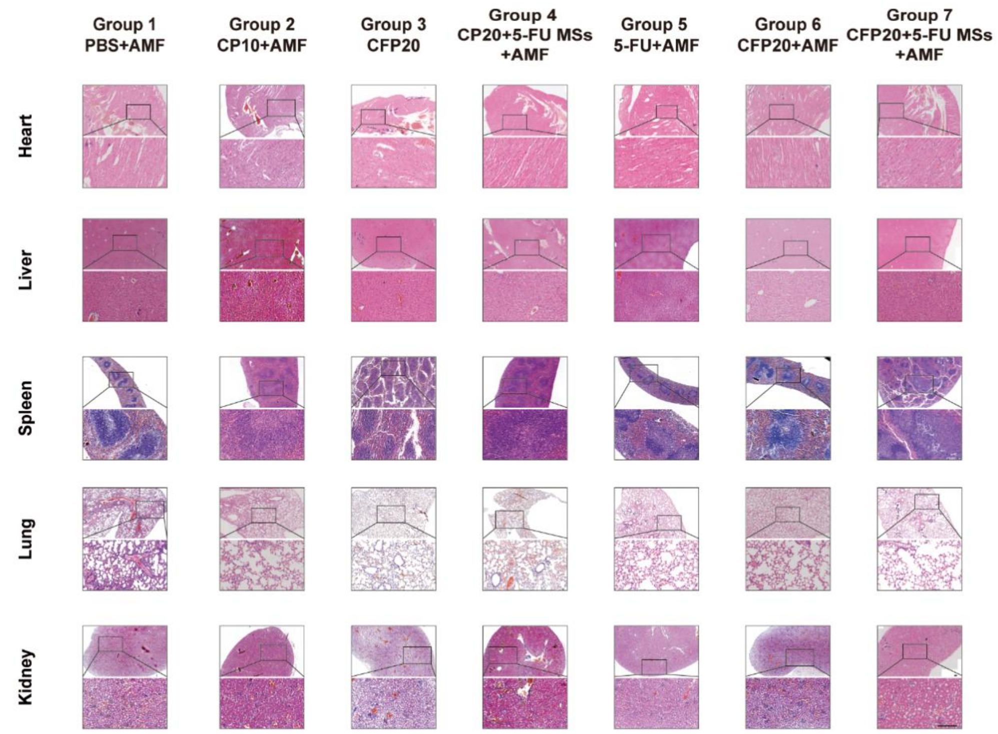

Supplementary Fig. 19 | In vivo establishment of the CDX model and the anti-tumor efficacy of HTHSG. Representative H&E staining images of major organs including heart, liver, spleen, lung, and kidney of each group in CDX tumor-bearing mice. Scale bar: 100 μm. Each experiment was repeated five times independently with similar results.

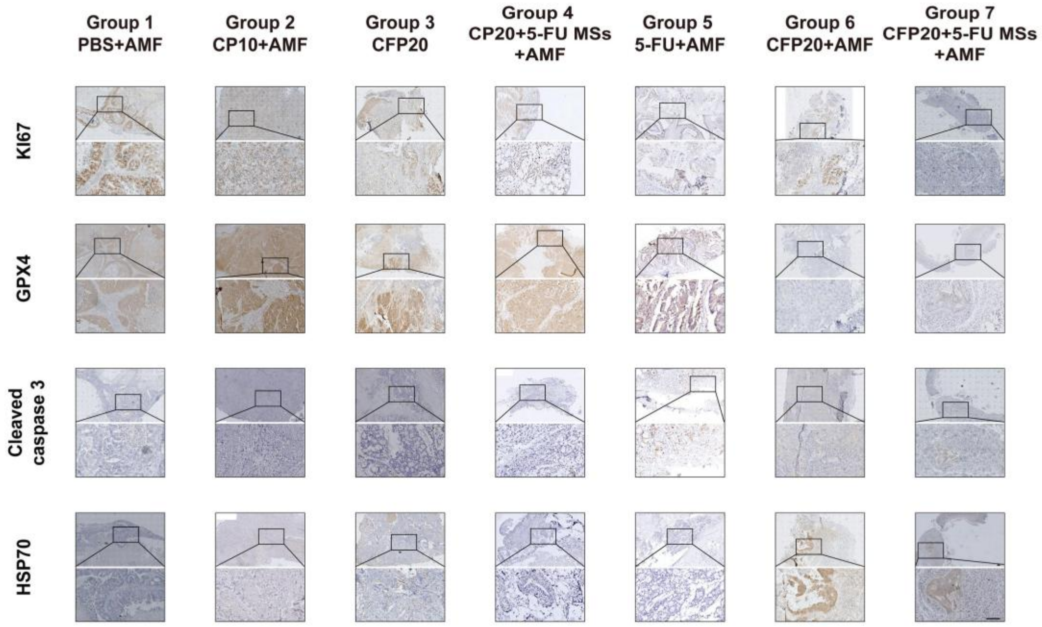

Supplementary Fig. 20 | In vivo anti-tumor efficacy and biocompatibility of HTHSG in a colorectal cancer PDX model. Immunohistochemical analysis of KI-67, Cleaved Caspase 3, GPX4, and HSP70 of PDX tumor slices in different groups. Scale bar: 100 μm. Each experiment was repeated four times independently with similar results.

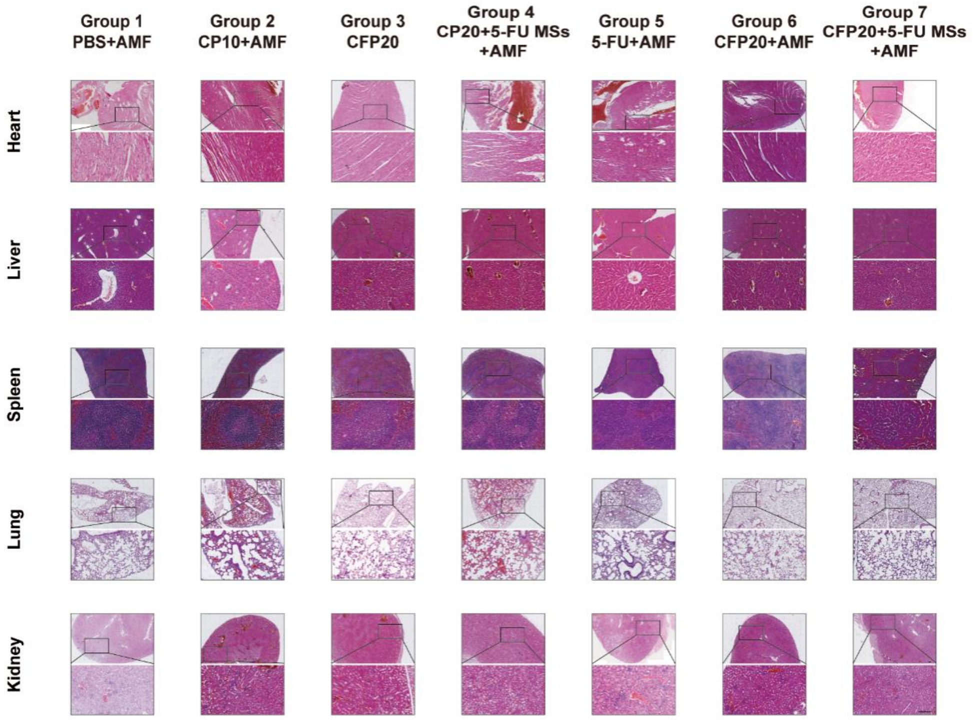

Supplementary Fig. 21 | In vivo anti-tumor efficacy and biocompatibility of HTHSG in a colorectal cancer PDX model. Representative H&E staining images of major organs including heart, liver, spleen, lung, and kidney of mice receiving PDX tumors from P1. Scale bar: 100 μm.

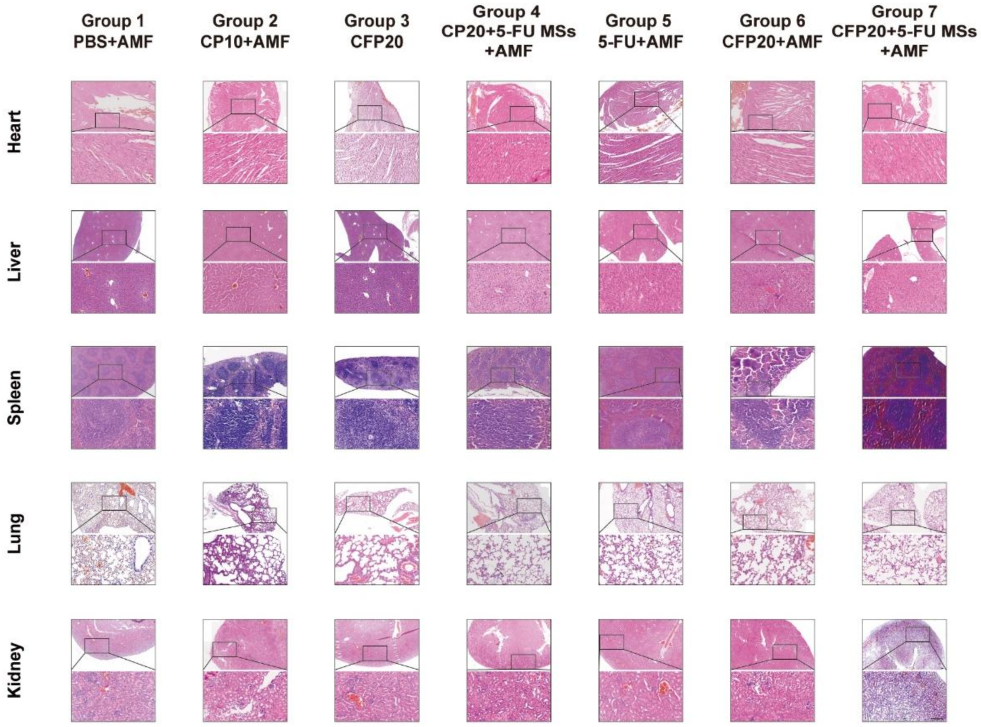

Supplementary Fig. 22 | In vivo anti-tumor efficacy and biocompatibility of HTHSG in a colorectal cancer PDX model. Representative H&E staining images of major organs including heart, liver, spleen, lung, and kidney of mice receiving PDX tumors from P2. Scale bar: 100 μm.

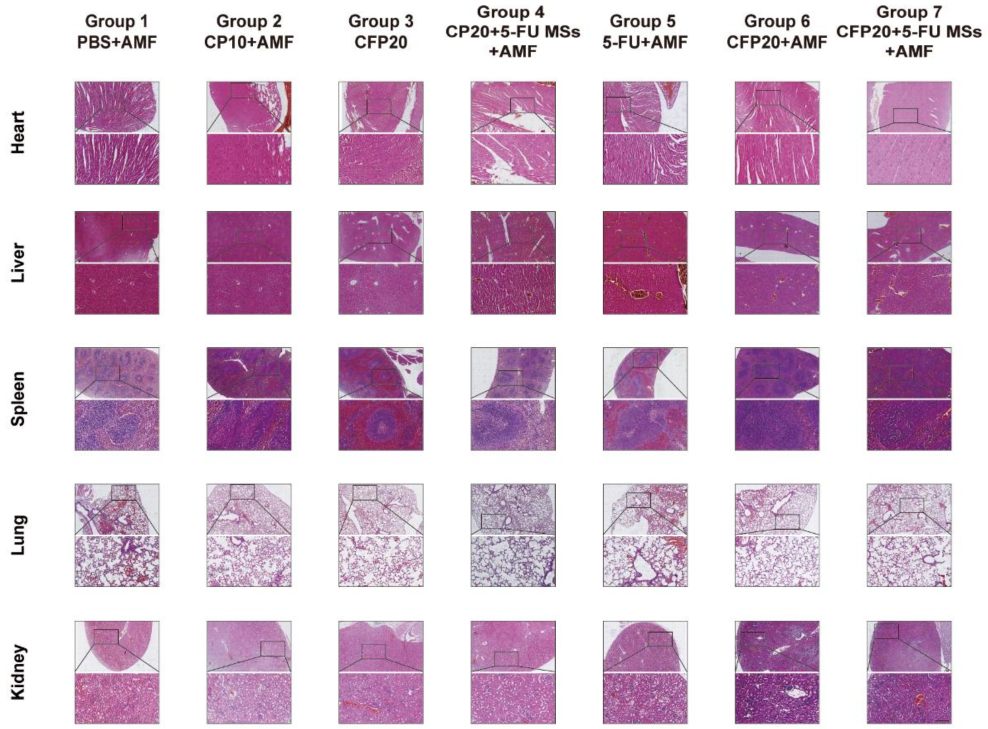

Supplementary Fig. 23 | In vivo anti-tumor efficacy and biocompatibility of HTHSG in a colorectal cancer PDX model. Representative H&E staining images of major organs including heart, liver, spleen, lung, and kidney of mice receiving PDX tumors from P3. Scale bar: 100 μm.

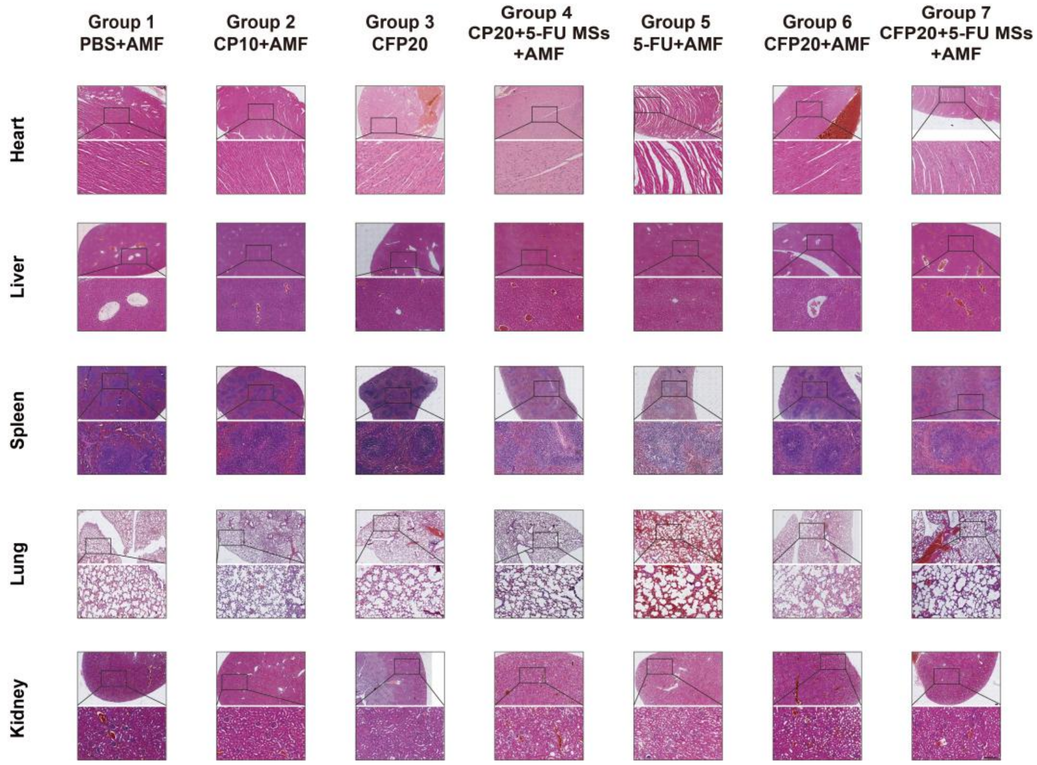

Supplementary Fig. 24 | In vivo anti-tumor efficacy and biocompatibility of HTHSG in a colorectal cancer PDX model. Representative H&E staining images of major organs including heart, liver, spleen, lung, and kidney of mice receiving PDX tumors from P4. Scale bar: 100 μm.

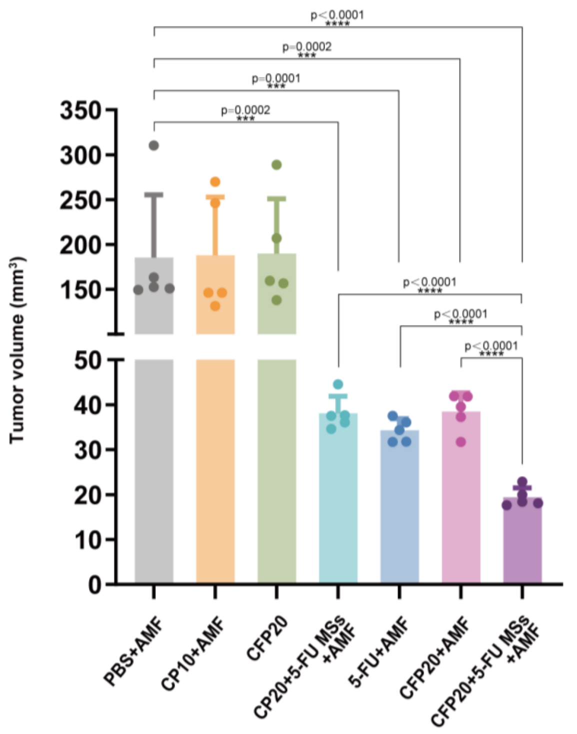

Supplementary Fig. 25 | Anti-tumor effects of HTHSG in orthotopic mouse models. Tumor volume of orthotopic mice models in PBS+AMF, CP10+AMF, CFP20, CP20-5-FU MSs+AMF, 5-FU+AMF, CFP20+AMF and CFP20+5-FU MSs+AMF groups. The data were presented as the mean ± SD (n = 5 mice per group). Statistical differences were analyzed by two-tailed unpaired Student's t-test. (ns, no significance, ***$p < 0.001$ and ****$p < 0.0001$)

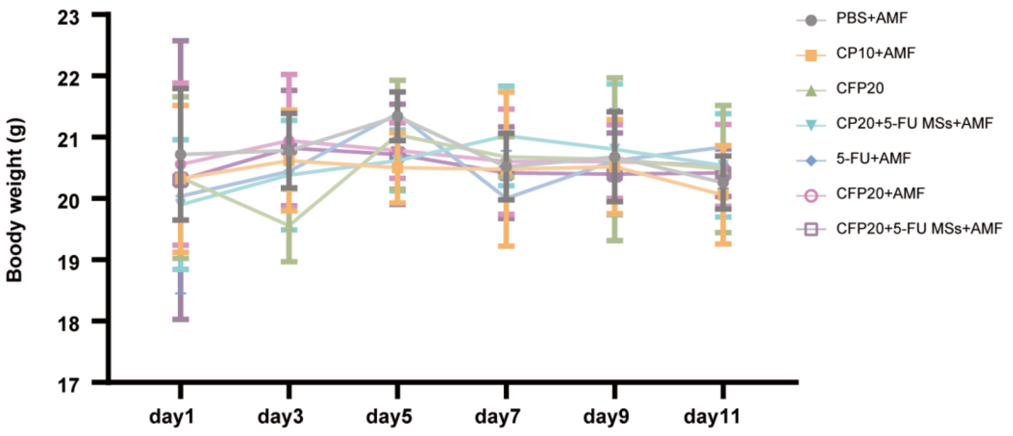

Supplementary Fig. 26 | Anti-tumor effects of HTHSG in orthotopic mouse models. Body weight changes across the different groups of orthotopic mouse models. The data were presented as the mean ± SD (n = 5 mice per group).

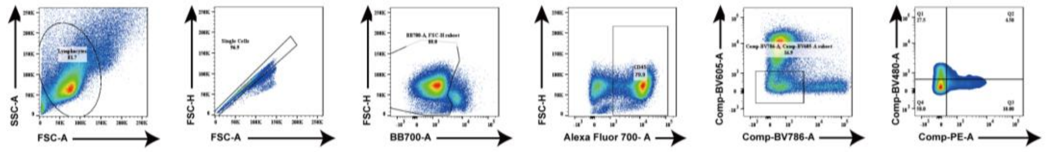

Supplementary Fig. 27 | Anti-tumor effects of HTHSG in orthotopic mouse models. Gating strategy for the identification of DCs in spleens.

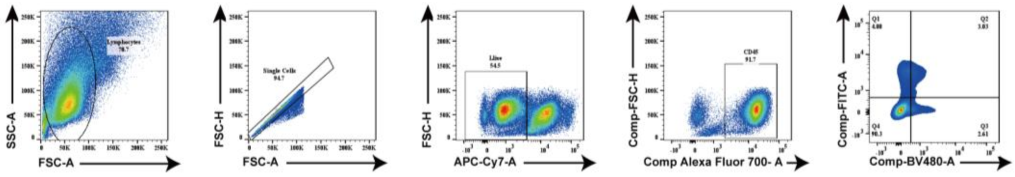

Supplementary Fig. 28 | Anti-tumor effects of HTHSG in orthotopic mouse models. Gating strategy for the identification of CD4+ T cells in spleens.

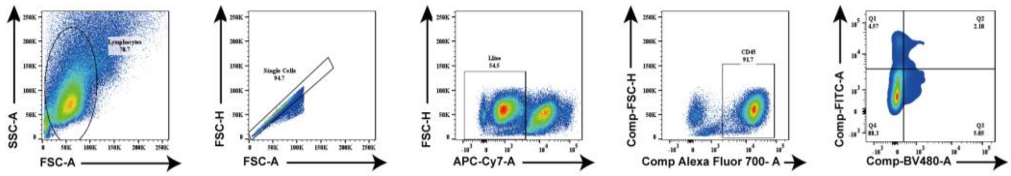

Supplementary Fig. 29 | Anti-tumor effects of HTHSG in orthotopic mouse models. Gating strategy for the identification of CD8+ T cells in spleens.

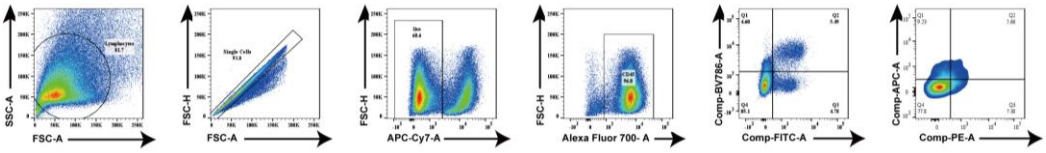

Supplementary Fig. 30 | Anti-tumor effects of HTHSG in orthotopic mouse models. Gating strategy for the identification of regulatory T cells in spleens.

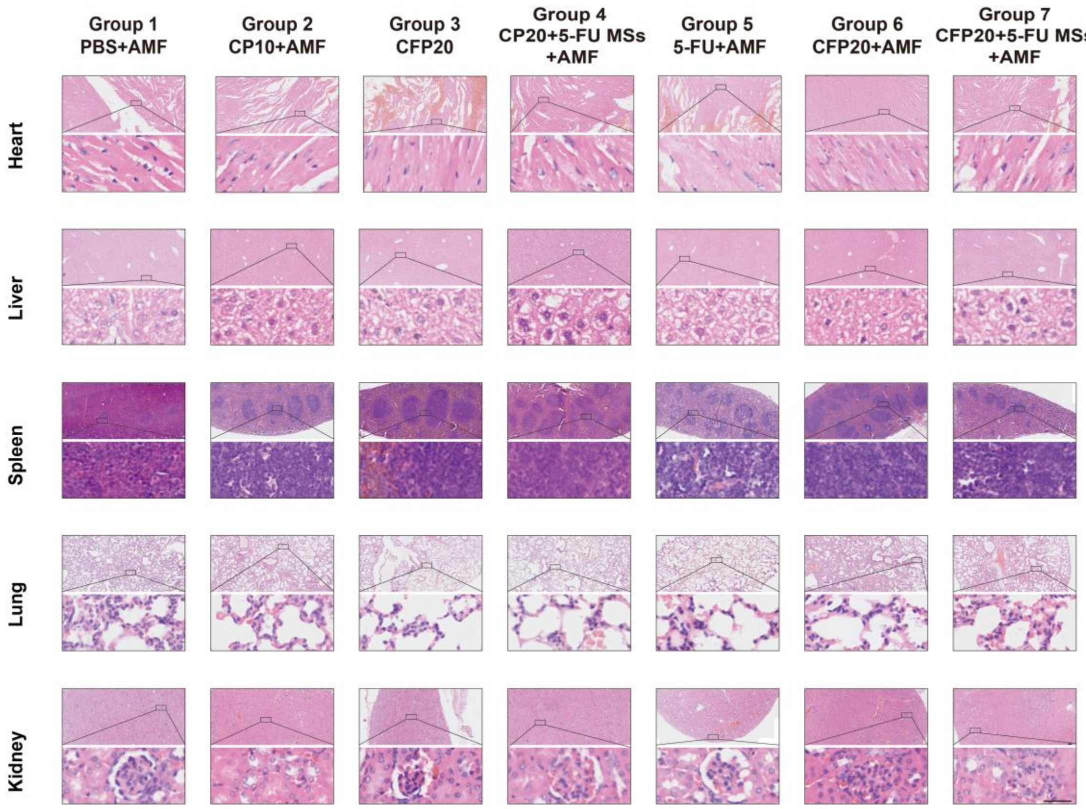

Supplementary Fig. 31 | Anti-tumor effects of HTHSG in orthotopic mouse models. Representative H&E staining images of major organs including heart, liver, spleen, lung, and kidney of each group in orthotopic mice models. Scale bar: 100 μm. Each experiment was repeated five times independently with similar results.

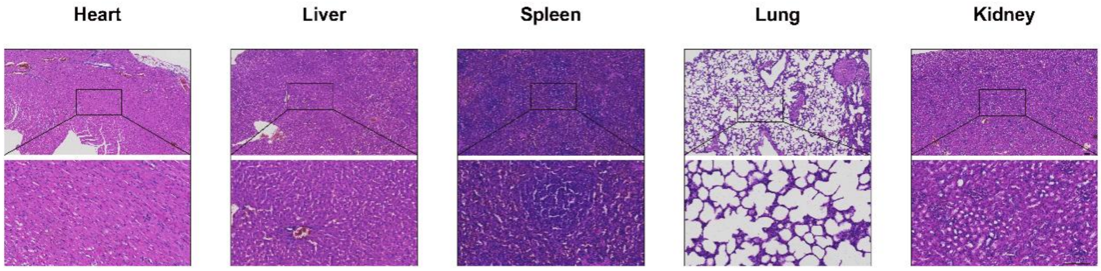

Supplementary Fig. 32 | Feasible application of HTHSG. Representative H&E staining images of major organs including heart, liver, spleen, lung, and kidney of rabbit. Scale bar: 100 μm

Supplementary Tables

Supplementary Table 1. Data comparison across CDX groups

Groups	Statements	Treatment methods	Tumor Volume (mm^3)	Tumor Weight (g)
Group 1	PBS+AMF group	Intraperitoneally injected with 100 μL PBS and exposed to AMF	685.8±387.0	0.5260±0.2483
Group 2	CP10+AMF group	Implanted subcutaneously with 25 mg CP10 hydrospongel around tumor and exposed to AMF	694.9±297.3	0.5180±0.1561
Group 3	CFP20 group	implanted subcutaneously with 25 mg CFP20 hydrospongel around tumor	670.5±300.3	0.5240±0.2541
Group 4	CP20+5-FU MSs +AMF group	Implanted subcutaneously with 25 mg CP20+5-FU MSs hydrospongel around tumor and exposed to AMF	169.2±54.34	0.2180±0.07662
Group 5	5-FU+AMF group	Intraperitoneally injected with 100 μL 5-FU solution and exposed to AMF	214.0±93.25	0.2540±0.09127
Group 6	CFP20+AMF group	Implanted subcutaneously with 25 mg CFP20 hydrospongel around tumor and exposed to AMF	247.8±217.4	0.2740±0.1479
Group 7	CFP20+5-FU MSs +AMF group	Implanted subcutaneously with 25 mg CFP20+5-FU MSs hydrospongel around tumor and exposed to AMF	56.20±47.88	0.04400±0.02074

Supplementary Table 2. Summary of patient information.

Patient number	4 (P1-P4)
Sex	3 Male, 1 Female
Cancer location	3 Rectal cancer, 1 Ascending colon cancer
Pathologic AJCC stage[1]	3 IIB, 1 IVA

Note: 1. AJCC Stages: American Joint Committee on Cancer (AJCC) TNM staging system.

Supplementary Table 3. Data comparison across PDX groups

Groups	Statements	Treatment methods	Tumor Volume (mm^3)				Tumor Weight(g)			
			**P1**	**P2**	**P3**	**P4**	**P1**	**P2**	**P3**	**P4**
Group 1	PBS+AMF group	Intraperitoneally injected with 100 μL PBS and exposed to AMF	**486**	**480**	**499.4525**	**245**	**0.7**	**0.8**	**0.8**	**0.4**
Group 2	CP10+AMF group	Implanted subcutaneously with 25 mg CP10 hydrospongel around tumor and exposed to AMF	**496.375**	**458.64**	**482.23**	**215.472**	**0.6**	**0.7**	**0.7**	**0.38**
Group 3	CFP20 group	Implanted subcutaneously with 25 mg CFP20 hydrospongel around tumor	**458.64**	**433.5**	**500**	**245**	**0.7**	**0.75**	**0.6**	**0.41**
Group 4	CP20+5-FU MSs +AMF group	Implanted subcutaneously with 25 mg CP20+5-FU MSs hydrospongel around tumor and exposed to AMF	**288**	**206.494**	**234**	**98.315**	**0.3**	**0.4**	**0.35**	**0.2**
Group 5	5-FU+AMF group	Intraperitoneally injected with 100 μL 5-FU solution and exposed to AMF	**202.3425**	**220.5**	**216**	**87.48**	**0.25**	**0.45**	**0.3**	**0.22**
Group 6	CFP20+AMF group	Implanted subcutaneously with 25 mg CFP20 hydrospongel around tumor and exposed to AMF	**191.896**	**194.208**	**245**	**84.27**	**0.3**	**0.35**	**0.25**	**0.12**
Group 7	CFP20+5-FU MSs +AMF group	Implanted subcutaneously with 25 mg CFP20+5-FU MSs hydrospongel around tumor and exposed to AMF	**48**	**62.5**	**18**	**18**	**0.1**	**0.1**	**0.04**	**0.02**

Supplementary Table 4. Data comparison across orthotopic mouse models

Groups	Statements	Treatment methods	Tumor Volume (mm^3)
Group 1	PBS+AMF group	Intraperitoneally injected with 100 μL PBS and exposed to AMF	172.3±213.2
Group 2	CP10+AMF group	Implanted directly to rectum with 25 mg CP10 hydrospongel around tumors through anus and exposed to AMF	175.8±149.5
Group 3	CFP20 group	Implanted directly to rectum with 25 mg CFP20 hydrospongel around tumors through anus	177.9±151.0
Group 4	CP20+5-FU MSs +AMF group	Implanted directly to rectum with 25 mg CP20+5-FU MSs hydrospongel around tumors through anus and exposed to AMF	38.40±6.693
Group 5	5-FU+AMF group	Intraperitoneally injected with 100 μL 5-FU solution and exposed to AMF	31.70±6.201
Group 6	CFP20+AMF group	Implanted directly to rectum with 25 mg CFP20 hydrospongel around tumors through anus and exposed to AMF	36.50±7.826
Group 7	CFP20+5-FU MSs +AMF group	Implanted directly to rectum with 25 mg CFP20+5-FU MSs hydrospongel around tumors through anus and exposed to AMF	18.90±2.012

Supplementary Table 5. AMF application conditions for different species

Species	Coil Diameter	Frequency	Field Strength	Time for HTHSG to reach 42℃	Exposure Period
**Mice**	5 cm	255 kHz	1.3 kA/m	Around 2 min	10 min
**Beagle**	16 cm	284 kHz	9.8 kA/m	Around 3-4 min	10 min
**Human (Dummy)**	32 cm	20 kHz	114.0 kA/m	Around 15 min	30 min

Supplementary Table 6. Comparation of hydrogels and nanoparticle delivery systems for treating colorectal cancer

				Stability of Material		Treatment Method			in vivo experiment				
Authors	Time	Foundational technologies	Method of Administration	Stability under Enzyme Induction	Physical Stability	Chemotherapy/ Drug treatment	Magnetothermal/ Photothermal Therapy	Chemodynamic Therapy	CDX	PDX	Orthotopic Model	Large Animal Model	Ref.
**Li L et al.**	2024	NPs[1] loaded hydrogel	Orally	√	√	√	×	√	√	×	√	×	67
**Fan R et al.**	2015	NPs loaded hydrogel	Injection	×	√	√	×	×	√	×	×	×	64
**Trombino S et al.**	2019	Hydrogel and MSs[2]	Orally	×	√	√	×	×	×	×	×	×	69
**Freitas de CF et al.**	2020	Hydrogel	Orally	×	√	√	×	×	×	×	×	×	63
**Shen MY et al.**	2019	MSs	Orally	√	√	√	√	×	√	×	√	×	15
**Pooresmaeil M et al.**	2019	Hydrogel	Orally	×	√	√	×	×	×	×	×	×	68
**Zhu X et al.**	2020	NPs	Injection	×	×	√	√	√	√	×	√	×	14
**Kim S et al.**	2023	Hydrogel	Injection	×	√	√	√	√	√	×	×	×	66
**Chen Y et al.**	2024	Hydrogel	Injection	×	×	√	×	×	√	×	√	×	62
**Ji Y et al.**	2023	NPs loaded hydrogel	Injection	×	√	×	√	×	×	×	×	×	65
**Zhang B et al.**	2024	NPs loaded hydrogel	Injection	×	√	√	×	×	×	×	×	×	70
**Our study**	2024	NPs loaded hydrogel and MSs	Local implantation	√	√	√	√	√	√	√	√	√	Our study

[1]NPs: nanoparticles; [2]MSs:microspheres

Supplementary Table 7. Detailed information on drug and compositions used in all animal studies

Animal stydies	Groups	Statements	Hydrospongel	Fe_3O_4@PDA NPs	5-FU	AMF
Mice (CDX/PDX/ Orthotopic Models)	Group 1	PBS+AMF group	-	-	-	Yes
	Group 2	CP10+AMF group	25 mg rectally	-	-	Yes
	Group 3	CFP20 group	25 mg rectally	About 10 mg	-	-
	Group 4	CP20+5-FU MSs +AMF group	25 mg rectally	-	About 1mg 5-FU in 25 mg hydrospongel	Yes
	Group 5	5-FU+AMF group	-	-	Total 1mg 5-FU intraperitoneally injected (0.5 mg per time)	Yes
	Group 6	CFP20+AMF group	25 mg rectally	About 10 mg	-	Yes
	Group 7	CFP20+5-FU MSs +AMF group	25 mg rectally	About 10 mg	About 1mg 5-FU in 25 mg hydrospongel	Yes
Beagle	-	CFP20+5-FU MSs (HTHSG) +AMF	150mg rectally	About 60 mg	About 6 mg 5-FU in 150 mg hydrospongel	Yes
