# Supplementary material for: A hollow-tube-like hydrospongel for multimodal therapy of advanced colorectal cancer
Source: Nat Commun. 2025 Aug 12;16:7464. doi: 10.1038/s41467-025-62880-x (PMC12343928; doi:10.1038/s41467-025-62880-x)
Supplement: Supplementary file 2 — Description of Additional Supplementary Files [file 41467_2025_62880_MOESM2_ESM.pdf]

Description of Additional Supplementary Files

Supplementary Movie 1 - Stability of CFP20 hollow tube under simulated bowel movement.

Supplementary Movie 2 - The process of HTHSG implantation and treatment in the CDX/PDX and Orthotopic models.

Supplementary Movie 3 - Feasible application of HTHSG in a beagle.
